# Delayed Diagnoses in Patients With Dizziness in the US Commonwealth of Virginia and the Tidewater Region

**DOI:** 10.1097/ONO.0000000000000046

**Published:** 2023-12-13

**Authors:** Kendra N. Walker, Kevin M. Guy, Peter G. Volsky

**Affiliations:** 1 Department of Otolaryngology, Eastern Virginia Medical School, Norfolk, Virginia.

**Keywords:** Dizziness, Vertigo, Vestibular disease, Vestibular dysfunction, Peripheral vestibular disease

## Abstract

**Objective:**

In a region of approximately 1.7 million people (Tidewater, coastal Virginia), identify secondary diagnoses in persons with dizziness.

**Methods:**

This cross-sectional study utilizing TriNetX included individuals in the region of interest diagnosed with dizziness between 2010 and 2020. Subsequent diagnoses of vestibular disease or medical conditions possibly associated with dizziness in the same subjects were catalogued.

**Results:**

During the study period, 31,670 subjects were identified with diagnoses of dizziness as a symptom; 18,390 subjects were subsequently given a dizziness-related nonvestibular diagnosis, and 930 were given a subsequent vestibular disease diagnosis. The proportion of subjects diagnosed with vestibular disease (3%) after the dizziness diagnosis is far below expected norms (25%–34%) in the general population. There were greater proportions of delayed diagnoses of labyrinth dysfunction (odds ratio [OR], 4.8; *P* < 0.0001), superior semicircular canal dehiscence (OR, 3.1; *P* = 0.0023), otolith disease (OR, 3.1; *P* = 0.0023), among others, and a decreased proportion of delayed diagnosis of benign paroxysmal positional vertigo (OR, 0.56; *P* < 0.0001).

**Conclusions:**

The discrepancy between expected and observed prevalence in our region indicates that vestibular disease is likely underdiagnosed.

Dizziness is a common chief complaint in primary care clinics and emergency departments (1–4). Evaluating the symptom of dizziness can be challenging for clinicians as the diagnosis relies heavily on the patient-reported history, during which patients often use the nonspecific term *dizziness* interchangeably with vertigo; moreover, patient responses are often inconsistent (2,5). The broad differential diagnosis of *dizziness* includes both vestibular and nonvestibular causes (1,2,6).

A recent study of regional prevalence in the Tidewater region of Virginia revealed that dizziness (symptom) was diagnosed in approximately 3% of patients, while vestibular disease (VD) was diagnosed in approximately 1% of patients (7). Given the substantial proportion of dizziness (symptom) diagnoses rendered without an underlying diagnosed medical cause in our region, we sought to identify temporally-associated subsequent diagnoses that could possibly relate to the dizziness symptom. Vestibular and nonvestibular diagnoses were considered, with the assumption that a second diagnosis following the symptom of dizziness indicated a potential cause of the symptom.

## METHODS

This study was approved by the Eastern Virginia Medical School institutional review board. Patient data for this study were obtained from the global health research network, TriNetX, containing deidentified clinical data for all patients seen at Sentara Healthcare facilities. Included in this cross-sectional study were adults between the ages 18 and 89 who have been diagnosed with the dizziness symptom (primary study cohort) at a Sentara Healthcare affiliated practice from January 1, 2010 to December 31, 2020. Data regarding sociodemographic information, diagnoses, and comorbidities were collected.

TriNetX was searched by International Classification of Diseases, 10th edition diagnosis codes for dizziness (symptom R42) and a variety of VD diagnoses and nonvestibular diagnoses (Table 1) within the proposed study period. From the population of all dizziness diagnoses (symptom R42), 2 separate cohorts were isolated: 1 cohort of VD diagnoses and 1 cohort of dizziness-related nonvestibular disorder (NVD) diagnoses, each occurring subsequent to a dizziness diagnosis by one or more days. The control group utilized in this study, for comparison to the cohort of VD diagnoses following a dizziness diagnosis, comprised of the total number of VD diagnosis in the region of interest (ROI) during the study period, as recently published in our related study (7).

**TABLE 1.** Comprehensive list of vestibular disease and nonvestibular disease diagnoses and associated ICD-10 codes included in the study population

Vestibular diagnosis	ICD-10	Nonvestibular diagnosis	ICD-10
Vestibular schwannoma	D33.3	Essential hypertension	I10
Meniere disease	H81.0	Hypertensive urgency	I16.0
Benign paroxysmal vertigo	H81.1	Orthostatic hypotension	I95.1
Vestibular neuronitis	H81.2	Mitral valve prolapse	I34.1
Otolith disease	H81.8X	Bradycardia	R00.1
Peripheral vestibulopathy	H81.9	Acute coronary syndrome	I24.0
Labyrinthine fistula	H83.1	Paroxysmal atrial fibrillation	I48.0
Labyrinthine dysfunction	H83.2X	Diastolic heart failure	I50.3
Superior semicircular canal dehiscence	H83.8X	Systolic heart failure	I50.2
Damage to vestibular nerve, initial	S04.60XA	Peripheral vascular disease	I73.9
Damage to vestibular nerve, subsequent	S04.60XD	Chronic ischemic heart disease	I25
Damage to vestibular nerve, sequela	S04.60XS	Acute myocardial infarction	I21
Damage to right vestibular nerve, initial	S04.61XA	Cerebral infarction	I63
Damage to right vestibular nerve, subsequent	S04.61XD	Intracranial hemorrhage	I62.9
Damage to right vestibular nerve, sequela	S04.61XS	Idiopathic intracranial hypertension	G93.2
Damage to left vestibular nerve, initial	S04.62XA	TIA	G45.9
Damage to left vestibular nerve, subsequent	S04.62XD	Migraine without aura	G43.0
Damage to left vestibular nerve, sequela	S04.62XS	Migraine with aura	G43.1
		Concussion	S06.0X
		Vestibular migraine	G43.809
		Seizure disorder	G40.909
		Wernicke-Korsakoff	F04
		Cerebellar ataxia	G11.9
		Vertigo of central origin	H81.4
		Motion sickness	T75.3XXA
		Nausea and vomiting	R11.2
		Brain lesion	G93.9
		Hydrocephalus	G91.9
		Head trauma	S09.90XA/S09.90XS/S09.90XD
		Altered mental status	R41.82
		Delirium	R41.0
		Dysautonomia	G90.1
		Chronic pain disorder	R52
		Sensorineural hearing loss, bilateral	H90.3
		Sensorineural hearing loss, unspecified	H90.5
		Anxiety disorders	F41
		Depressive episode	F32
		Somatoform disorder	F45.9
		Hyperthyroidism	E05.90
		Hypothyroidism, unspecified	E03.9
		T2DM	E11
		T1DM	E10
		Menopause	Z78.0
		Dyspnea	R06.00
		Acute respiratory failure	J96.00
		Sleep apnea	G47.3
		Allergic rhinitis	J30.9
		Asthma	J45
		Sepsis	A41.9
		Aminoglycoside ototoxicity	H91.09/H91.01/H91.02/H91.03
		Other ototoxicity	H93.8x9/H93.8x1/H93.8x2/H93.8x3
		Morbid obesity	E66.01
		Rheumatoid arthritis	M06.9
		Fibromyalgia	M79.7

ICD-10 indicates International Classification of Diseases, 10th edition; TIA, transient ischemic attack.

Chi-squared tests were performed to analyze the observed differences between groups. Odds ratios (ORs) and *P* values were calculated for the prevalence of VD diagnoses subsequent to dizziness (symptom R42) compared to all diagnoses of VD in the database for the study period (control). An OR was considered significant if the value was not close to 1. We used a 95% confidence interval with a *P* value <0.05 signifying statistical significance.

### Region of Interest (ROI)

Prior work (7) outlined the assumptions and limitations of TriNetX as a source and appraisal of the subject selection methodology. No substantial changes in the population have occurred during the interim to alter rationale for utilizing TriNetX to study the ROI.

## RESULTS

### Study Sample

Demographic data for this population are outlined in Table 2.

**TABLE 2.** Demographics of patients who initially received a diagnoses for dizziness followed by a dizziness-related nonvestibular disease or vestibular disease diagnosis

Diagnosis	Dizziness later diagnosed with nonvestibular disease	Dizziness later diagnosed with vestibular disease
Demographics		
Frequency	18,390	930
Mean age	65	66
Standard deviation	16	15
Gender		
Female, %	63	70
Male, %	37	30
Ethnicity		
Non-Hispanic, %	95	93
Hispanic, %	3	4
Other, %	2	3
Race		
White, %	66	66
Black, %	29	26
Other, %	5	8

### Dizzy Patients Later Receiving a Contributing Nonvestibular Diagnosis

During the 10-year study period, there were 31,670 diagnoses of dizziness (symptom R42) (7).

We identified 18,390 subjects (58.1%) in which a subsequent diagnosis of NVD was rendered, plausibly accounting for the dizziness symptom. These individuals did not receive any VD diagnoses during the study period. NVD diagnosed subsequent to a dizziness diagnosis, and their frequencies, are outlined in Table 3. The most common NVD included essential hypertension (63%), diabetes mellitus type II (25%), anxiety disorder (21%), chronic ischemic heart disease (17%), and depressive episode (16%). Sensorineural hearing loss was diagnosed infrequently (6%).

**TABLE 3.** Frequencies of nonvestibular diagnoses following initial dizziness diagnosis between 2010 and 2020, grouped by organ system

Subsequent nonvestibular diagnosis	ICD-10 code	%
Cardiovascular		
Essential hypertension	I10	63
Hypertensive urgency	I16.0	1
Orthostatic hypotension	I95.1	8
Mitral valve prolapse	I34.1	1
Bradycardia	R00.1	5
Acute coronary syndrome	I24.0	<1
Paroxysmal atrial fibrillation	I48.0	9
Diastolic heart failure	I50.3	8
Systolic heart failure	I50.2	8
Peripheral vascular disease	I73.9	7
Chronic ischemic heart disease	I25	17
Acute myocardial infarction	I21	3
Cerebrovascular		
Cerebral infarction	I63	6
Intracranial hemorrhage	I62.9	<1
Idiopathic intracranial hypertension	G93.2	<1
TIA	G45.9	4
Neurologic		
Migraine without aura	G43.0	4
Migraine with aura	G43.1	2
Concussion	S06.0X	1
Vestibular (or other variant) migraine	G43.809	1
Seizure disorder	G40.909	2
Wernicke-Korsakoff	F04	0
Cerebellar ataxia	G11.9	0
Vertigo of central origin	H81.4	1
Motion sickness	T75.3XXA	<1
Nausea and vomiting	R11.2	10
Brain lesion	G93.9	<1
Hydrocephalus	G91.9	<1
Head trauma	S09.90XA/S09.90XS/S09.90XD	2
Altered mental status	R41.82	7
Delirium	R41.0	2
Dysautonomia	G90.1	<1
Chronic pain disorder	R52	6
Sensorineural hearing loss, bilateral	H90.3	4
Sensorineural hearing loss, unspecified	H90.5	2
Psychiatric		
Anxiety disorders	F41	21
Depressive episode	F32	16
Somatoform disorder	F45.9	<1
Endocrine/reproductive		
Hyperthyroidism	E05.90	1
Hypothyroidism, unspecified	E03.9	14
T2DM	E11	25
T1DM	E10	2
Menopause	Z78.0	4
Respiratory		
Dyspnea	R06.00	10
Acute respiratory failure	J96.00	3
Sleep apnea	G47.3	13
Allergic rhinitis	J30.9	10
Asthma	J45	11
Toxic/metabolic/trauma		
Sepsis	A41.9	4
Aminoglycoside ototoxicity	H91.09/H91.01/H91.02/H91.03	<1
Other ototoxicity	H93.8x9/H93.8x1/H93.8x2/H93.8x3	1
Morbid obesity	E66.01	14
Other		
Rheumatoid arthritis	M06.9	2
Fibromyalgia	M79.7	4

ICD-10 indicates International Classification of Diseases, 10th edition; TIA, transient ischemic attack.

### Dizzy Patients Later Receiving a Vestibular Disease Diagnosis

We identified 930 subjects (2.94%) in which a subsequent VD diagnosis was rendered. The array of subsequent VD diagnoses is outlined in Table 4. In order of decreasing frequency, these were benign paroxysmal positional vertigo (BPPV) (n = 680, 73%), labyrinthine dysfunction (n = 110, 12%), Meniere disease (n = 100, 11%), vestibular schwannoma (n = 70, 8%), peripheral vestibulopathy (n = 60, 6%), vestibular neuronitis (n = 30, 3%), otolith disease (n = 10, 1%), and superior semicircular canal dehiscence (n = 10, 1%).

**TABLE 4.** Frequencies of vestibular disease diagnoses following initial dizziness diagnosis between 2010 and 2020

Subsequent VD diagnosis	ICD-10 code	Frequency	%
Benign neoplasm of cranial nerve			
Vestibular schwannoma	D33.3	70	8
Meniere disease	H81.0	100	11
Benign paroxysmal positional vertigo	H81.1	680	73
Vestibular neuronitis	H81.2	30	3
Other disorders of vestibular function			
Otolith disease	H81.8X	10	1
Unspecified disorder of vestibular function			
Peripheral vestibulopathy	H81.9	60	6
Labyrinthine fistula	H83.1	0	0
Labyrinthine dysfunction	H83.2X	110	12
Other specified diseases of inner ear			
Superior semicircular canal dehiscence	H83.8X	10	1
Damage to vestibular nerve, initial	S04.60XA	0	0
Damage to vestibular nerve, subsequent	S04.60XD	0	0
Damage to vestibular nerve, sequela	S04.60XS	0	0
Damage to right vestibular nerve, initial	S04.61XA	0	0
Damage to right vestibular nerve, subsequent	S04.61XD	0	0
Damage to right vestibular nerve, sequela	S04.61XS	0	0
Damage to left vestibular nerve, initial	S04.62XA	0	0
Damage to left vestibular nerve, subsequent	S04.62XD	0	0
Damage to left vestibular nerve, sequela	S04.62XS	0	0

ICD-10 indicates International Classification of Diseases, 10th edition; VD, vestibular disease.

#### Comparison Between Subsequent VD and Total VD

This population can be compared to the total population of all VD diagnoses rendered during the study period, using findings from our recently published-related study that identified 8480 total VD diagnoses in the ROI (7). The most common diagnoses from this population were BPPV (n = 7040, 83%), vestibular schwannoma (n = 580, 7%), Meniere disease (n = 360, 4%), vestibular neuronitis (n = 280, 3%), labyrinthine dysfunction (n = 230, 3%), peripheral vestibulopathy (n = 150, 2%), otolith disease (n = 30, <1%), and superior semicircular canal dehiscence (n = 30, <1%).

**FIG. 1.**
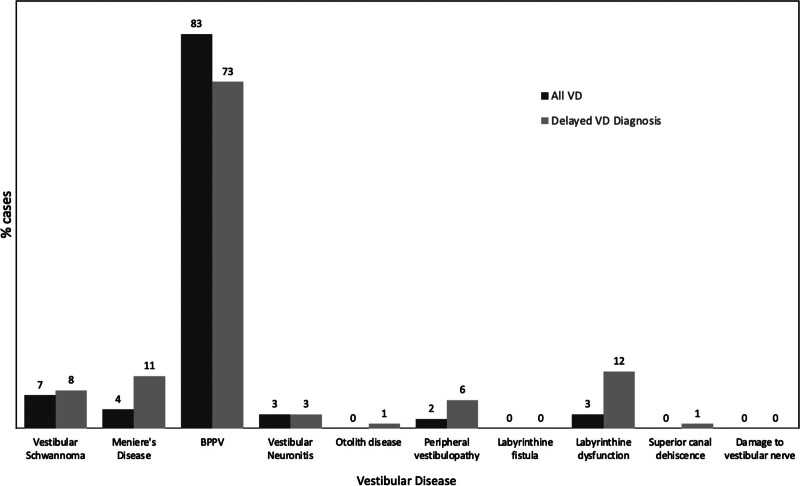
Disease frequencies from the total population of primary VD diagnoses compared with disease frequencies of VD diagnoses subsequent to a dizziness diagnosis. This bar graph displays a greater proportion of Labyrinthine dysfunction, Meniere disease, vestibular schwannoma, and peripheral vestibulopathy diagnosed subsequent to the dizziness symptom, and a decreased proportion of BPPV diagnosed subsequent to the dizziness symptom, compared to all diagnoses of primary VD. BPPV indicates benign paroxysmal positional vertigo; VD, vestibular disease.

Subsequent diagnoses of VD comprise 11% (930/8480) of the overall VD diagnoses during the study period. The principal differences are depicted in Figure 1, including increased proportions of subsequent diagnoses of labyrinthine dysfunction (OR, 4.8; *P* < 0.0001), peripheral vestibulopathy (OR, 3.8; *P* < 0.0001), superior semicircular canal dehiscence (OR, 3.1; *P* = 0.0023), otolith disease (OR, 3.1; *P* = 0.0023), and Meniere disease (OR, 2.7; *P* < 0.0001), when compared with the total population of VD diagnoses during the study period. In contrast, BPPV (OR, 0.56; *P* < 0.0001) is less often diagnosed subsequent to dizziness, as it is a primary diagnosis.

## DISCUSSION

Our research (7) is the first to quantify burdens of dizziness and VD in the region. Delayed or missed diagnoses of dizziness can magnify physical and mental disability (8,9). Most cases of dizziness do not receive a subsequent diagnosis that could account for the symptom. The present study identifies key differences in the population of patients who receive a diagnosis of VD or NVD subsequent to a diagnosis of dizziness and provides a critical comparison against all patients with dizziness and VD.

Concerning VD alone, the conditions diagnosed in greater proportion after assignment of the dizziness symptom (R42) are chronic otologic conditions, typically diagnosed by a specialist. A retrospective analysis of patients referred to a specialized center reported fewer diagnoses of unclear dizziness from 70% to 10% upon referral, validating the assumption that specialists are less likely to apply the dizziness symptom diagnosis (10). Consequently, subjects with dizziness who are subsequently diagnosed with VD (about 3%) plausibly reflect the proportion of individuals accessing specialist care to comprehensively evaluate and manage the dizziness symptom.

Comparing the cohort of all subjects with VD and the cohort of subjects with dizziness receiving a subsequent diagnosis of VD (Fig. 1), we found that patients with BPPV tend to receive this as a primary diagnosis, rather than a diagnosis subsequent to a prior encounter for dizziness. This can likely be explained by the ability to diagnose BPPV using the Dix-Hallpike test, a physical exam positioning maneuver that can be performed in any setting with a bed, sofa, table, or stretcher (11–14). Other peripheral vestibular disorders, typically diagnosed subsequent to dizziness, require more nuanced physical examination—such as the observation of spontaneous nystagmus, the head impulse test, the head-shake test—or videonystagmography for diagnosis (3,14–16).

In accordance with existing literature (17–19), VD was found to be more common in females compared to males, and there was a slightly greater proportion of females in the group with diagnoses of subsequent VD (70%) compared to the group with subsequent NVD (63%), as demonstrated in Table 2. Still, the frequency of females with subsequent VD diagnoses falls near the reported 66.7% predominance of all VD diagnoses in females, as seen in an epidemiologic survey of 70 million patients published by Hülse et al (17). Frequencies for age, race, and ethnicities did not show any substantial differences between the 2 groups.

Previous studies estimate that vestibular disorders account for 25%–34% of dizziness presentations (20,21). In this study, they were only identified in about 3% of dizziness cases. The incidence of VD diagnoses following a dizziness diagnosis in our ROI falls vastly short of the expected true prevalence of VD in patients with dizziness. In the ROI, perhaps VD is underdiagnosed, or alternatively, a preponderance of dizziness is attributable to NVD. Notably, no subsequent diagnosis was identified in 12,350 of the 31,670 dizzy subjects, or possibly, the symptom resolved.

In cases of acute vertigo, a complete neurological examination and supportive care may be appropriate for primary care settings. Notably, visiting emergency departments for complaints of dizziness can introduce unnecessary costs for patients, due to the overuse of neuroimaging that is insufficient in diagnosing peripheral causes of dizziness (4,22,23). Admittedly, arriving at one diagnosis or several diagnoses to account for the symptom of dizziness is a challenge. After a general medical evaluation, referral to specialized care providers, such as neurologists, cardiologists, psychiatrists, otolaryngologists, or vascular specialists, could increase diagnostic rates of chronic dizziness and vertigo.

### Selection Bias Inherent to Study Design.

The database used in this study, TriNetX, introduced limitations in data collection and analysis. To offer large amounts of data from the entire Sentara Healthcare System, the platform compresses data and reports of all frequencies rounded to the nearest factor of 10 and all percentage values rounded to the nearest whole number. This reporting error should not impact the interpretation of the results due to the large size of the population explored in this study. Additionally, due to the size of the population receiving subsequent diagnoses of NVD, data were compressed by the TriNetX database, reporting only the frequency and percent values for prevalence of disease from 9820 of 18,390 total diagnoses in the cohort. Enough diagnoses are included in the reported percentage values that these should be generalizable to the whole population and are, therefore, reported in this study. Because frequencies for demographics were only reported in percentages, numerical frequency reported in the results was calculated based on the percentage value of the whole population number, and is, therefore, also a rounded value.

We also consider the limitation that some dizziness diagnoses may have been rendered near the end of the study period and are, therefore, excluded from the cohorts of patients who received a follow-up diagnosis to explain their dizziness. Therefore, this study may report an underestimated number of patients who received a follow-up diagnosis and may overestimate the number of patients who never received an additional diagnosis following a diagnosis of dizziness. However, the criteria to be included in the subsequent diagnoses cohort only required a minimum of 1 day between diagnoses; therefore, few cases were likely excluded. It was only possible to query TriNetX for this one or more day criteria, the database does not provide the duration of the interval between dizziness diagnoses and subsequent diagnoses for neither individual cases nor an average for the cohort. The TriNetX database did not enable us to collect data on frequency of patient consultation or referral to a specialist in our study population. Additionally, we chose not to investigate resolved dizziness in this study, since physicians do not always remove symptoms from problem lists in medical records, lending to overestimation of prevalence.

## CONCLUSIONS

A majority of dizziness symptom diagnoses were followed by plausible (nonvestibular) causes (58%), while very few individuals with the dizziness symptom diagnoses were ultimately diagnosed with VD (3%). Many individuals with the dizziness symptom (39%) did not receive an additional qualifying diagnosis to explain the dizziness symptom.

One may surmise that a majority of individuals either experience spontaneous resolution of dizziness (improbable) or their symptom is incompletely evaluated (probable); comparisons to existing prevalence data (7) in the ROI reveal that VD is either very uncommon (improbable) or underdiagnosed (probable). We consider it important to deepen our understanding of these regional differences, because consequences of delayed or missed diagnoses include lost opportunities for early access to medical management or rehabilitation and prolonged disability.

## ACKNOWLEDGMENTS

We thank the HADSI staff for assisting with the development of the research methods and statistical analysis of the data included in this manuscript.

## FUNDING SOURCES

This research did not receive any funding from public, commercial, or not-for-profit sources.

## CONFLICT OF INTEREST STATEMENT

None declared.

## DATA AVAILABILITY STATEMENT

The datasets generated during and/or analyzed during the current study are publicly available.
